# IL-37 and IL-36 Cytokine Profiles in Chronic Hepatitis Delta During Bulevirtide Therapy

**DOI:** 10.3390/pathogens15020198

**Published:** 2026-02-10

**Authors:** Verdiana Zulian, Martina De Sanctis, Silvia Pauciullo, Roberta Sciamanna, Eleonora Cimini, Paola Del Porto, Anna Rosa Garbuglia

**Affiliations:** 1 Virology Laboratory, National Institute for Infectious Diseases Lazzaro Spallanzani IRCCS, 00149 Rome, Italy; verdiana.zulian@inmi.it (V.Z.); martina.desanctis@inmi.it (M.D.S.); roberta.sciamanna@inmi.it (R.S.); annarosa.garbuglia@inmi.it (A.R.G.); 2 Laboratory of Cellular Immunology and Pharmacology, National Institute for Infectious Diseases Lazzaro Spallanzani IRCCS, 00149 Rome, Italy; eleonora.cimini@inmi.it; 3 Department of Biology and Biotechnology “Charles Darwin”, Sapienza University of Rome, 00100 Rome, Italy; paola.delporto@uniroma1.it

**Keywords:** hepatitis delta virus, antiviral treatment, bulevirtide, cytokines, IL-36, IL-37, biomarkers

## Abstract

Chronic hepatitis delta is the most severe form of viral hepatitis and is associated with rapid progression to cirrhosis and hepatocellular carcinoma. Although bulevirtide (BLV) effectively inhibits hepatitis D virus (HDV) entry, immunological biomarkers reflecting treatment response and residual viral activity remain poorly defined. This study investigated the serum profiles of interleukin-37 (IL-37) and IL-36 isoforms (IL-36α, IL-36β, and IL-36γ) in 22 HBV/HDV-coinfected patients receiving BLV monotherapy (2 mg/day). Serum cytokine levels were measured by ELISA at baseline (BL) and after 48 weeks of BLV treatment (TW48) and compared with HBV-monoinfected patients under nucleos(t)ide-analogue therapy and healthy donors. Patients were stratified according to virological, biochemical, and combined responses. At both BL and TW48, serum IL-37, IL-36α, and IL-36β levels were significantly higher in HBV/HDV-coinfected patients than in comparison groups (all *p* < 0.05), independent of treatment response, indicating a persistent cytokine signature during BLV therapy. IL-36β levels significantly decreased over time, particularly in biochemical non-responders (*p* = 0.0469), whereas IL-36α remained elevated and differed at TW48 between combined responders and non-responders (*p* = 0.0400). IL-36γ was detectable only in a small subset of patients. Notably, in a subgroup of patients evaluated at week 96, baseline IL-37 levels were significantly lower in those achieving virological response compared with non-responders (*p* = 0.0275). Moreover, IL-37 was the only cytokine showing a significant positive correlation with HDV RNA levels at TW48 when quantified by the AltoStar^®^ assay (*p* = 0.033; R^2^ = 0.7563). Overall, HBV/HDV-coinfected patients display a distinct IL-37/IL-36 cytokine profile during BLV therapy. The association between IL-37 and residual viremia supports further investigation of this cytokine as a complementary biomarker for monitoring low-level viral activity during treatment.

## 1. Introduction

Hepatitis delta virus (HDV) is an enveloped virus with a single-stranded circular RNA genome. It is also defined as a satellite virus of hepatitis B virus (HBV), which provides HBsAg protein required for HDV virion assembly. Its genome is 1.7 Kb in length and encodes a single protein, the HDV antigen (HDAg), which exists in two isoforms: the small antigen (S-HDAg), which promotes HDV replication and the large antigen (L-HDAg), which interacts with HBsAg during virion assembly and has an inhibitory effect on HDV replication. It has been estimated that 5% of patients with chronic HBV infection (corresponding to about 20 million individuals) are coinfected with HDV [1]. HDV infection can occur through two distinct modes: coinfection and superinfection. Coinfection occurs when both HBV and HDV infect the patient simultaneously. It is characterized by the presence of anti-Hepatitis B core antigen (anti-HBc) IgM together with the appearance of anti-HDV IgM. In contrast, superinfection occurs when HDV infects a patient with chronic HBV infection and is characterized by the absence of anti-HBc IgM. In cases of coinfection, both HBV and HDV are cleared in approximately 95% of cases [2]. In contrast, in cases of superinfection, HDV persists in about 80% of patients and may promote cirrhosis development within an average of five years, as well as increase the risk of liver cancer within ten years [3,4]. Overall, among HBV carriers, one fifth of liver cirrhosis are linked to HDV coinfection, and one sixth of hepatocellular carcinoma (HCC) cases occur in HBV-HDV-coinfected individuals [3,5]. HDV coinfection increases profibrogenic factors such as transforming growth factor (TGF-β) and enhances pro-inflammatory responses [6,7,8,9]. By inducing oxidative stress, HDV also disrupts mitochondrial homeostasis, leading, among other effects, the activation of Signal Transducer and Activator Transcription-3 (STAT-3) [10]. The rapid progression to cirrhosis in chronic HDV infection requires prompt therapeutic intervention. Until the last decade, pegylated interferon-alpha (PEG-IFNα) was the only antiviral treatment against HDV. Patients with chronic HDV infection and detectable HDV RNA, with or without advanced fibrosis or cirrhosis, were eligible for treatment. HDV RNA clearance was observed in 20–30% of treated patients [11]. However, late relapse occurred in about 40% of patients within 5–8 years after antiviral treatment suspension [12,13]. This high relapse rate may be partly explained by the limited sensitivity of the tests used to detect HDV RNA, allowing low-level residual viremia to remain undetected and causing the relapse of HDV RNA [14]. Recently, bulevirtide (BLV), an entry inhibitor of HBsAg into hepatocyte, appears to be more effective for the treatment of chronic HDV infection. BLV is a lipopeptide mimicking myristoylated pre-S1 (amino acid 2-48) domain of HBV and interferes with HBV and HDV viral entry by binding sodium taurocholate cotransporting polypeptide (NTCP), which is the entry receptor for both HBV and HDV [15]. BLV treatment was approved by the European Medical Agency (EMA) in 2020 and Agenzia Italiana del Farmaco (AIFA) in 2023. The drug is administered concomitantly with nucleos(t)ide analogues (NUC) anti-HBV therapy and it is well tolerated. At week 48, real-world studies report biochemical, virological, and combined response rates of 61–69%, 64–65%, and 44–51%, respectively, with BLV 2 mg/day [16,17]. Currently, BLV therapy must be maintained as virological and clinical responses have been shown to increase progressively over time. Furthermore, a validated stopping rule to ensure treatment discontinuation without subsequent relapse has yet to be established. The absence of HDV RNA for up to 96 weeks of treatment has not been a reliable marker of cure. However, undetectable HDV RNA does not exclude the future development of HCC [18], nor does it eliminate the risk of viral rebound following discontinuation of BLV therapy [19].

Parameters that describe the progression of an infection, and that may therefore be considered by physicians and researchers when developing effective therapeutic strategies, include cytokines and chemokines levels. In fact, these mediators are key elements in the regulation of host immunity and play essential role in controlling and ultimately eliminating pathological processes [20]. Recently, a subset of cytokines belonging to the interleukin-1 (IL-1) superfamily has received increasing attention for its involvement in the interplay between pathologies and immune response. The IL-1 superfamily includes 11 proteins with either pro-inflammatory (IL-1α, IL-1β, IL-18, IL-33, IL-36α, IL-36β, and IL-36γ) or anti-inflammatory (IL-1Ra, IL-36Ra, IL-38, and IL-37) activities [21]. Among these cytokines, IL-37, officially named only in 2010 [22], and IL-36, discovered approximately 20 years ago [23], remain under active investigation.

IL-36 is made up of four isoforms, IL-36α, IL-36β, IL-36γ, and IL-36 receptor antagonist (IL-36Ra). During inflammation IL-36 expression increases in monocytes/macrophages, keratinocytes, and epithelial cells [24]. Once matured, IL-36 binds IL-36R and IL-1RAcP, inducing receptor dimerization and triggering a signaling cascade involving MyD88, MAPKs, NF-kB, and STAT3 [25]. This signaling leads to the production of factors such as VEGF-A, ICAM-1, and VCAM-1 [26], along with cytokines and chemokines including IL-1β, TNFα, CCL20, CXCL1, CXCL2, IL-8, G-CSF, IL-17C and, indirectly throughout this last cytokine, IL-17A [27,28]. Several studies about the IL-36 and IL-37 cytokine profiles are reported in HIV, SARS-CoV-2, IAV, HSV-1, and HBV-infected patients [20,29,30,31,32,33,34,35]. However, analogous data in HBV/HDV-coinfected patients remain extremely limited, and studies specifically investigating IL-37 and the four isoforms of IL-36 in this subgroup of patients are even more scarce.

Therefore, this study aims to analyze the expression of serum IL-37 and IL-36 isoforms in HBV/HDV patients undergoing BLV treatment from baseline (BL) through week 48 (TW48).

## 2. Materials and Methods

### 2.1. Study Population

Twenty-two patients with chronic HBV/HDV coinfection treated with BLV were enrolled in this study between October 2023 and May 2024. All patients received BLV monotherapy (2 mg/day) for at least 48 weeks; among them, 16 patients reached 96 weeks of treatment. Thirteen patients had cirrhosis (59% of patients), while nine patients did not reach this stage of hepatological damage (41% of patients) at the beginning of the study. Three patients tested positive for anti-HCV antibodies, although they were negative for HCV RNA for 10 years. The control group consisted of five healthy donors (HD) randomly selected with no history of hepatological disease recorded. Concurrently, we included ten HBV-monoinfected patients under NUC treatment to exclude that the serological levels of IL-37 and IL-36 isoforms observed in our cohort were attributable to HBV infection. None of the HBV-monoinfected patients had hepatocellular carcinoma, and only one patient had cirrhosis. Elevated transaminase levels (>40 U/L) were observed in only one patient. All HBV-monoinfected patients were HBeAg negative and anti-HBe positive. Serum samples were collected from all HBV/HDV-coinfected patients at BL and at treatment weeks 48 for clinical, virological, and immunological evaluation. Serological markers (anti-HD IgM, anti-HD IgG, HBsAg, anti-HBc IgG, HBcrAg) were tested at both time points, alongside IL-37, IL-36α, IL-36β, IL-36γ, ALT, AST, albumin, bile acids, total bilirubin, and platelet count. Meanwhile, HBeAg, anti-HBe, and anti-HBc IgG and IgM were only tested at BL. The HDV genotype and subtype were evaluated by sequencing as a reported in our previous work [36]. All patients continued to receive their prescribed HBV NUC treatment. All experiments were performed in accordance with the Declaration of Helsinki. A virological response was defined as a ≥2 Log decline in HDV RNA or when HDV RNA was undetectable or below the lower limit of detection (LLOD). Furthermore, biochemical response was defined as ALT normalization (ALT < 40 U/L).

### 2.2. Serological Markers

The DIAPRO assay was used to detect anti-HD IgG and IgM following the manufacturer’s instructions (DIAPRO, Milan, Italy). HBsAg, antibodies to HBsAg (anti-HBs), HBeAg and antibodies to HBeAg (anti-HBe) were assessed using with the chemiluminescent microparticle enzyme assay with Alinity instrument (Abbott Diagnostics, Abbott Park, IL, USA). HBcrAg serum levels were evaluated using the automated Lumipulse G600II system (Fujirebio Europe, Gent, Belgium). The Lumipulse G600II was also used to evaluate the levels of the IgG anti-HBc with the Lumipulse^®^ G Anti-HBc IgG-N assay (Fujirebio, Tokyo, Japan). Results were reported as a cut-off index (COI). HBV DNA and HDV RNA were tested respectively with COBAS 6800 System (Roche Applied Science, Basel, Switzerland), and Bosphore HDV RNA kit v1.0 (Low-Limit of Detection, LLOD 10 IU/mL) (Anthalia, Istanbul, Turkey). In addition, HDV RNA was also assessed using the AltoStar^®^ HDV RT-PCR Kit (Altona Diagnostics GmbH, Hamburg, Germany) (LLOD 1 IU/mL).

### 2.3. IL-37, IL-36α, IL-36β, and IL-36γ Detection

Serum samples were obtained from peripheral blood, after centrifugation at 3500× *g* rpm for 20 min and immediately stored at −80 °C. Levels of the cytokines IL-37, IL-36α (IL14F6), IL-36β (IL1F8) and IL-36γ (IL1F9) were measured using sandwich ELISA assays kit of Cusabio^®^, following the manufacturer’s instructions. The detection range of the above kits is 31.25–2000 pg/mL, 15.6–1000 pg/mL, 6.25–400 pg/mL, and 78–5000 pg/mL respectively. The spectrophotometer used to read the ELISA plates is BioTek Instruments© “Synergy HTX Multimode reader” which uses the company’s software, Gen5 Microplate Reader and Imager Software (Version 3.02).

### 2.4. Statistical Analysis

Continuous variables were expressed as medians and interquartile ranges (IQRs) or ranges, while categorical variables were reported as frequencies and percentages. Comparisons between two independent groups were performed using the Mann–Whitney U test and paired comparisons were assessed using the Wilcoxon signed-rank test. Cytokine levels were analyzed according to cirrhosis status, biochemical response, virological response, and combined response. Longitudinal changes in cytokine concentrations were evaluated by calculating delta values (Δ), defined as the difference between TW48 and BL (TW48−BL). Associations between serum cytokine levels and HDV RNA concentrations were evaluated using Spearman’s rank correlation, followed by linear regression analysis. All statistical tests were two-tailed, and a *p*-value < 0.05 was considered statistically significant. Statistical analyses were performed using GraphPad Prism 9 software (GraphPad Software, Inc., La Jolla, CA, USA) and RStudio version 2024.09.0 + 375 (RStudio, Boston, MA, USA).

## 3. Results

### 3.1. Study Population

A total of 22 patients with chronic HBV/HDV coinfection, who received BLV monotherapy (2 mg/day) for at least 48 weeks, were included in this study. The median age of BLV-treated patients was 50 (range: 35–68) years, 50% were male, and the median body mass index (BMI) was 24.5 (IQR: 23.0–29.7) kg/m^2^. Sixteen patients (72.7%) had previously received interferon-based therapy, and cirrhosis was present in 59.1% of the cohort. All 22 patients were infected with HDV genotype 1 (19 with subtype 1e, 2 with subtype 1b, and 1 with subtype 1c), as previously reported [36].

At BL, patients showed median ALT and AST concentrations of 87.5 (IQR: 55.3–103.8) U/L and 72.0 (IQR: 52.3–88.5) U/L, respectively. Biochemical parameters included albumin at a median value of 4.2 (IQR: 4.0–4.4) g/dL, bile acids at 11.7 (IQR: 6.4–18.9) µmol/L, and total bilirubin at 0.7 (IQR: 0.7–0.9) mg/dL. The median platelet count was 113.5 (IQR: 90.1–190.0) × 10^3^/µL and median alpha-Fetoprotein (AFP) was 4.9 (IQR: 3.5–6.7) ng/mL. At BL, 36.4% (8/22) of patients had AFP levels > 6 ng/mL, whereas at TW48 all patients showed normalized AFP values (<6 ng/mL). With respect to virological parameters, HDV RNA levels had a median of 4.9 (IQR: 3.9–5.9) Log cp/mL, while HBV DNA was detectable in 4.6% (1/22) of individuals. Median concentrations of HBsAg, HBcrAg, and anti-HBc IgG were 3.6 (IQR: 3.3–4.1) Log IU/mL, 3.4 (IQR: 2.6–4.1) Log U/mL, and 58.5 (IQR: 40.8–264.2) cut-off index (COI), respectively. Characteristics of the 22 HBV/HDV-coinfected patients at BL are shown in Table 1.

After 48 weeks of BLV treatment, virological, biochemical, and combined responses were achieved by 54.5% (12/22), 68.2% (15/22), and 50.0% (11/22) of patients, respectively. In the group of 10 patients with HBV-monoinfected patients included as a control, median age was 51 (range: 47–63) years and 60% were male. Median HBsAg and HBV DNA levels were 3.8 (IQR: 3.2–4.2) Log IU/mL and 1923.0 (IQR: 324.0–4947.5) IU/mL, respectively.

### 3.2. Longitudinal Analysis of Serum IL-37, IL-36α, IL-36β, and IL-36γ in HBV/HDV-Coinfected Patients Treated with Bulevirtide

**Figure 1:**
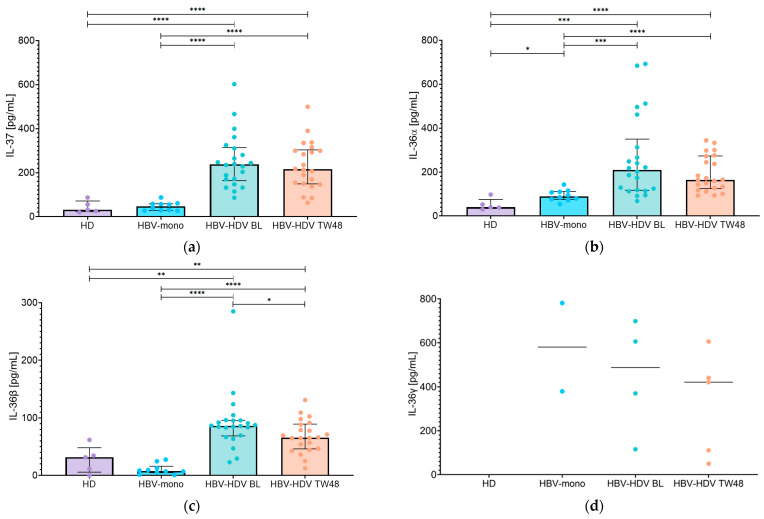
Serum levels of IL-37 and IL-36 family cytokines in HBV/HDV-coinfected patients. Serum concentrations of (**a**) IL-37, (**b**) IL-36α, (**c**) IL-36β, and (**d**) IL-36γ were measured in healthy donors (HD), HBV-monoinfected patients (HBV-mono), and HBV/HDV-coinfected patients (HBV-HDV) at baseline (BL) and after 48 weeks of bulevirtide treatment (TW48). Individual data points are shown, with bars representing the median and interquartile range (IQR). *p*-values are indicated as follows: <0.0332 (*), <0.0021 (**), <0.0002 (***), <0.0001 (****).

Overall, at BL, serum IL-37 median levels (Figure 1a) were significantly higher in HBV/HDV-coinfected patients compared with HBV-monoinfected patients (236.9 [IQR: 163.8–313.9] pg/mL vs. 46.7 [IQR: 28.1–58.5] pg/mL; *p <* 0.0001). A similar pattern was observed at TW48, with persistently higher IL-37 levels in HBV/HDV-coinfected patients compared with HBV-monoinfected patients (215.0 [IQR: 148.8–303.6] pg/mL vs. 46.7 [IQR: 28.1–58.5] pg/mL; *p* < 0.0001). Although IL-37 levels showed an overall decreasing trend over the 48-week treatment period (Figure 1a) in the HBV/HDV-coinfected group, this reduction was not statistically significant (*p* > 0.05). All comparisons between HBV/HDV-coinfected patients and healthy donors were statistically significant at both BL and TW48 (median IL-37 value in healthy donors: 31.1 [IQR: 22.7–71.1] pg/mL; all *p* < 0.05).

The same analysis performed for IL-36α (Figure 1b) revealed significantly higher serum levels in HBV/HDV-coinfected patients compared with HBV-monoinfected patients (209.9 [IQR:116.8–350.0] vs. 89.6 [IQR: 75.5–111.3] pg/mL, *p* = 0.0002). This difference persisted at TW48, with IL-36α levels in HBV/HDV-coinfected patients (164.6 [IQR: 124.7–273.1] pg/mL) remaining significantly elevated relative to HBV-monoinfected patients (*p* < 0.0001). Over the 48-week BLV treatment period, IL-36α levels showed a tendency to decrease in the HBV/HDV-coinfected group (Figure 1b); however, this variation was not statistically significant. Moreover, IL-36α levels in HBV/HDV-coinfected patients differed significantly from those observed in healthy donors at both BL and TW48 (all *p* < 0.05).

Consistent with the findings observed for IL-37 and IL-36α, baseline evaluation of IL-36β serum levels (Figure 1c) revealed significantly higher concentrations in HBV/HDV-coinfected patients (86.2 [IQR: 68.8–95.8] pg/mL) compared with HBV-monoinfected patients (7.7 [IQR: 4.9–16.0] pg/mL, *p* < 0.0001). This difference was preserved at TW48, with IL-36β levels in HBV/HDV-coinfected patients (65.8 [IQR: 46.2–89.3] pg/mL) remaining significantly elevated compared with those in HBV-monoinfected patients (*p* < 0.0001) (Figure 1c). Over the 48-week BLV treatment period, IL-36β levels showed a significant decrease in the HBV/HDV-coinfected group (*p* = 0.0391). Across both time points, IL-36β levels measured in HBV/HDV-coinfected patients were consistently distinct from those of healthy donors (31.5 [IQR: 5.8–48.2] pg/mL; all *p* < 0.05).

Regarding IL-36γ, levels were predominantly below the assay’s lower limit of detection of the assay (LoD: 39 pg/mL) across all study groups (Figure 1d). IL-36γ concentrations were undetectable in all healthy donors. Among HBV-monoinfected patients, detectable IL-36γ levels were observed in only two individuals (379.5 and 781.0 pg/mL), while the remaining samples were below the LoD. In HBV/HDV-coinfected patients, IL-36γ was detectable only in a limited subset of individuals at both BL and TW48 (Figure 1d). Specifically, detectable IL-36γ levels were observed in four patients at BL (487.9 [IQR: 179.3–675.0] pg/mL) and in five patients at TW48 (421.0 [IQR: 80.1–523.1] pg/mL), with only three patients showing detectable levels at both time points.

Given the potential impact of advanced liver disease on cytokine profiles, IL-37, IL-36α, IL-36β, and IL-36γ levels were additionally evaluated according to cirrhosis status. Serum IL-37 levels were comparable between cirrhotic and non-cirrhotic HBV/HDV-coinfected patients at both BL and TW48. Specifically, at BL (Figure S1a), median IL-37 levels were 228.8 [IQR: 152.0–287.4] pg/mL in cirrhotic patients and 243.8 [IQR: 158.1–380.4] pg/mL in non-cirrhotic patients. At TW48 (Figure S1b), IL-37 levels were 235.3 [IQR: 147.9–307.5] pg/mL and 212.8 [IQR: 119.2–314.9] pg/mL in cirrhotic and non-cirrhotic patients, respectively. In both groups, IL-37 levels remained largely stable over the 48-week treatment period, with no statistically significant changes observed.

Similarly, serum IL-36α levels did not differ significantly between cirrhotic and non-cirrhotic patients at either BL or TW48. At BL, median IL-36α levels were 221.2 [IQR: 119.2–503.9] pg/mL in cirrhotic patients and 173.3 [IQR: 106.2–244.3] pg/mL in non-cirrhotic patients (Figure S1a), while at TW48 median values were 183.3 [IQR: 146.1–287.1] pg/mL and 133.6 [IQR: 114.5–204.6] pg/mL in cirrhotic and non-cirrhotic patients, respectively (Figure S1b). Overall, IL-36α levels tended to decrease over the 48-week treatment period, although no statistically significant temporal changes were observed.

A comparable pattern was observed for IL-36β. In fact, no statistically significant differences were detected between cirrhotic and non-cirrhotic patients at either BL or TW48. Specifically, at BL, cirrhotic patients had a median IL-36β level of 90.8 [IQR: 65.2–100.5] pg/mL, whereas non-cirrhotic patients had a median value of 84.7 [IQR: 76.4–91.6] pg/mL (Figure S1a). At TW48, IL-36β median levels were 66.3 [IQR: 45.2–83.7] pg/mL in cirrhotic patients and 65.3 [IQR: 45.5–94.8] pg/mL in non-cirrhotic patients (Figure S1b). Across the 48-week treatment period, IL-36β levels decreased in both cirrhotic and non-cirrhotic patients; however, these reductions did not reach statistical significance.

Consistent with the overall analysis, IL-36γ was detectable only in a very limited number of cirrhotic and non-cirrhotic HBV/HDV-coinfected patients, precluding formal statistical comparisons between groups. Among cirrhotic patients, detectable IL-36γ levels were observed in two individuals at BL (370.2 and 115.6 pg/mL) and in two individuals at TW48 (605.6 and 440.7 pg/mL). In non-cirrhotic patients, IL-36γ was detectable in three patients at BL (605.6 [IQR: 234.6–698.2] pg/mL) and in four patients at TW48 (132.2 [IQR: 64.9–354.2] pg/mL). Of note, in the three non-cirrhotic patients with detectable IL-36γ levels at both BL (Figure S1a) and TW48 (Figure S1b), IL-36γ concentrations decreased over time.

### 3.3. Changes in IL-37, IL-36α, IL-36β, and IL-36γ in Bulevirtide Treated HBV/HDV-Coinfected Patients According to Biochemical Response

**Figure 2:**
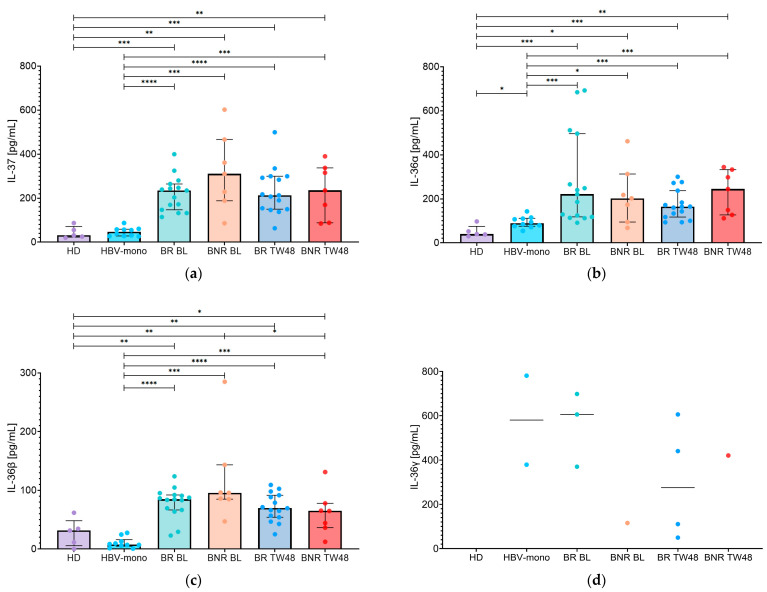
Serum levels of IL-37 and IL-36 family cytokines stratified by biochemical response. Serum concentrations of (**a**) IL-37, (**b**) IL-36α, (**c**) IL-36β, and (**d**) IL-36γ were measured in healthy donors (HD), HBV-monoinfected patients (HBV-mono), and HBV/HDV-coinfected patients stratified according to biochemical response into biochemical responders (BR) and biochemical non-responders (BNR) at baseline (BL) and after 48 weeks of bulevirtide treatment (TW48). Individual data points are shown, with bars representing the median and interquartile range (IQR). *p*-values are indicated as follows: <0.0332 (*), <0.0021 (**), <0.0002 (***), <0.0001 (****).

When the HBV/HDV-coinfected group was stratified according to biochemical response, biochemical responders (BR) at BL showed IL-37 levels (Figure 2a) that were higher than HBV-monoinfected patients (234.6 [IQR: 146.8–264.5] pg/mL vs. 46.7 [IQR: 28.1–58.5] pg/mL; *p* < 0.0001). Similarly, biochemical non-responders (BNR) had higher levels of IL-37 (310.3 [IQR: 188.4–466.4] pg/mL) compared with HBV-monoinfected patients (46.7 [IQR: 28.1–58.5] pg/mL; *p* = 0.0002). At TW48, IL-37 levels (Figure 2a) remained elevated in both BR and BNR patients compared with HBV-monoinfected patients (212.8 [IQR: 149.7–299.7] pg/mL and 235.3 [IQR: 88.0–337.2] pg/mL, respectively; *p* < 0.0001 and *p* = 0.0002). Furthermore, no significant differences in IL-37 levels were observed between BR and BNR patients at either BL or TW48 (Figure 2a). Finally, over the 48-week BLV treatment period, IL-37 levels showed a tendency toward reduction in BNR patients, while remaining largely stable in BR patients; however, no statistically significant intra-group changes were observed.

**Figure 3:**
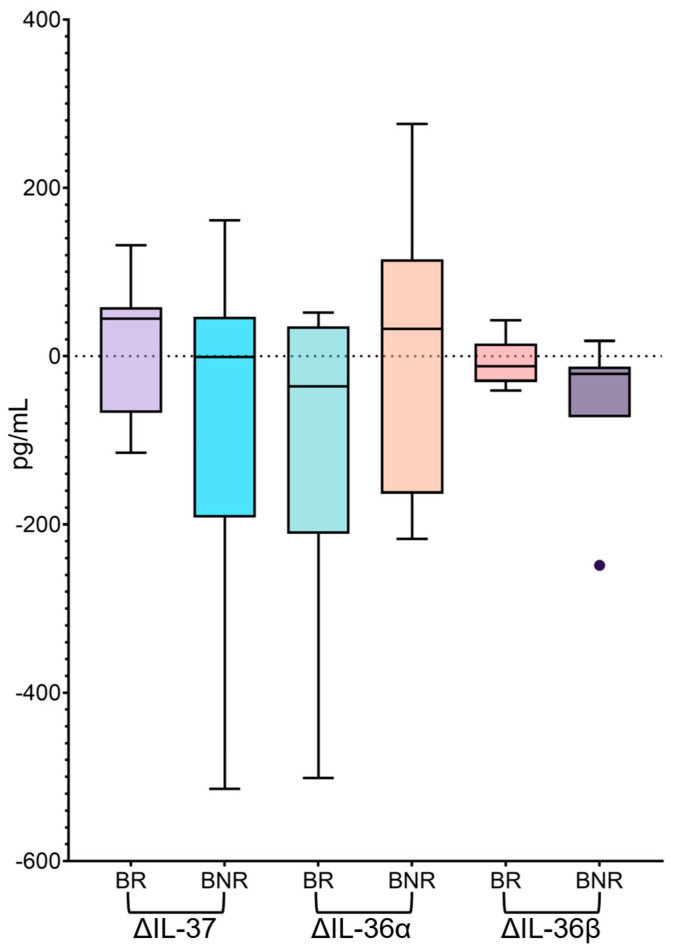
Changes (Δ) in serum IL-37 and IL-36 cytokine levels according to biochemical response. Box plots show the change (Δ) in serum concentrations of IL-37, IL-36α, and IL-36β between baseline (BL) and week 48 of bulevirtide treatment (TW48) in HBV/HDV-coinfected patients, stratified as biochemical responders (BR) and biochemical non-responders (BNR). Δ values were calculated as TW48−BL. Boxes indicate the interquartile range (IQR) with the median; whiskers extend to 1.5 × IQR.

To further characterize longitudinal changes, IL-37 delta values (TW48−BL) were evaluated according to biochemical response. Consistently, delta analyses highlighted marked inter-individual variability, with a positive median ΔIL-37 in BR patients (44.4 [IQR: −67.3 to 58.1] pg/mL) and ΔIL-37 values centered around zero in BNR patients (−1.1 [IQR: −191.9 to 46.9] pg/mL), without significant differences between subgroups (Figure 3).

A similar distribution was observed for IL-36α levels at BL (Figure 2b). In fact, IL-36α levels were higher in both BR and BNR patients compared with HBV-monoinfected patients (221.2 [IQR: 117.9–496.2] pg/mL and 202.1 [IQR: 94.6–312.8] pg/mL, respectively; *p* = 0.0002 and *p* = 0.0330). This pattern persisted at TW48 (Figure 2b), with IL-36α levels remaining elevated in both BR (164.4 [IQR: 117.5–273.3] pg/mL; *p* = 0.0007) and BNR patients (244.5 [IQR: 127.1–332.9] pg/mL; *p* = 0.0007) compared with HBV-monoinfected patients. Direct comparison between BR and BNR patients did not reveal statistically significant differences in IL-36α levels at either BL or TW48; nevertheless, at TW48, BR patients tended to display lower IL-36α levels than BNR patients (Figure 2b). IL-36α levels showed divergent trends over the treatment period, with a tendency toward reduction in BR patients, whereas levels in BNR patients remained largely stable, although these changes were not statistically significant.

Evaluation of IL-36α delta values revealed divergent trajectories based on biochemical response (Figure 3). In BR patients, ΔIL-36α was predominantly negative (−35.8 [IQR: −211.2 to 35.5] pg/mL), consistent with an overall tendency toward reduction. Conversely, BNR patients showed a slightly positive median ΔIL-36α (32.6 [IQR: −163.5 to 115.1] pg/mL), suggesting largely stable levels with a mild upward shift in a subset of individuals.

At BL, BR showed higher IL-36β levels (84.7 [IQR: 66.6–92.1] pg/mL) than HBV-monoinfected patients (*p* < 0.0001). A similar finding was observed among BNR, who also exhibited elevated IL-36β levels at BL (95.7 [IQR: 85.0–143.4] pg/mL) compared with HBV-monoinfected patients (*p* = 0.0001). At TW48 (Figure 2c), IL-36β levels remained significantly higher in both BR and BNR patients compared with HBV-monoinfected patients (69.6 [IQR: 54.1–91.6] pg/mL and 64.8 [IQR: 36.4–77.9] pg/mL, respectively; *p* < 0.0001 and *p* = 0.0007). Although IL-36β levels did not differ significantly between BR and BNR patients at BL or TW48 (Figure 2c), a significant reduction from BL to TW48 was observed in BNR patients (*p* = 0.0469), while the decrease in BR patients did not reach statistical significance.

Consistently, delta analysis of IL-36β indicated an overall downward shift in both biochemical subgroups (Figure 3). Median ΔIL-36β values were negative in both BR (−11.9 [IQR: −30.7 to 15.1] pg/mL) and BNR patients (−21.2 [IQR: −72.6 to −12.2] pg/mL). Notably, in BNR patients the interquartile range of ΔIL-36β values remained entirely below zero, supporting a more consistently negative change over time compared with BR.

Finally, IL-36γ remained largely undetectable in both BR and BNR at both time points (Figure 2d). At BL, detectable IL-36γ levels were observed in three BR patients (605.6 [IQR: 370.2–698.2] pg/mL) and in only one BNR patient (115.7 pg/mL). At TW48, IL-36γ was detectable in four BR patients (275.6 [IQR: 64.88–564.3] pg/mL) and in one BNR patient (421.0 pg/mL), while all remaining samples were below the lower limit of detection (Figure 2d). Due to the limited number of detectable values, no formal statistical comparisons were performed between BR and BNR patients.

### 3.4. Changes in IL-37, IL-36α, IL-36β, and IL-36γ in Bulevirtide Treated HBV/HDV-Coinfected Patients According to Virological and Combined Response

**Figure 4:**
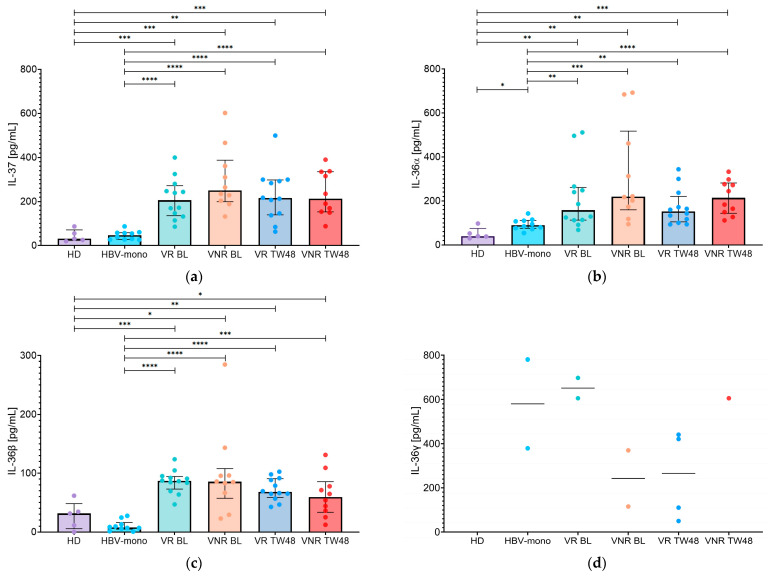
Serum levels of IL-37 and IL-36 family cytokines stratified by virological response. Serum concentrations of (**a**) IL-37, (**b**) IL-36α, (**c**) IL-36β, and (**d**) IL-36γ were measured in healthy donors (HD), HBV-monoinfected patients (HBV-mono), and HBV/HDV-coinfected patients stratified according to virological response into virological responders (VR) and virological non-responders (VNR) at baseline (BL) and after 48 weeks of bulevirtide treatment (TW48). Individual data points are shown, with bars representing the median and interquartile range (IQR). *p*-values are indicated as follows: <0.0332 (*), <0.0021 (**), <0.0002 (***), <0.0001 (****).

A similar analysis of IL-37 was performed after stratification according to virological response (Figure 4a). At BL, serum IL-37 levels were significantly higher in both virological responders (VR) and non-responders (VNR) compared with HBV-monoinfected patients (205.5 [IQR: 135.8–271.9] pg/mL and 249.5 [IQR: 199.6–387.6] pg/mL, respectively; both *p* < 0.0001). This difference was maintained at TW48, with IL-37 levels remaining significantly elevated in both VR (215.0 [IQR: 139.5–297.9] pg/mL) and VNR patients (212.7 [IQR: 152.8–335.6] pg/mL) compared with HBV-monoinfected patients (both *p* < 0.0001). Moreover, no significant differences were observed between VR and VNR patients at either BL or TW48.

At BL, VR presented higher IL-36α levels (157.5 [IQR: 112.7–261.6] pg/mL) than HBV-monoinfected patients (*p* = 0.0071). VNR similarly exhibited elevated IL-36α levels (219.5 [IQR: 159.5–517.3] pg/mL) compared with HBV-monoinfected patients (*p* = 0.0002). At TW48 (Figure 4b), IL-36α levels remained increased in both VR (151.6 [IQR: 104.8–221.0] pg/mL) and VNR patients (213.9 [IQR: 143.8–281.6] pg/mL) relative to HBV-monoinfected groups (*p* = 0.0034 and *p* < 0.0001, respectively). No statistically significant differences were detected between VR and VNR patients at either BL or TW48; however, IL-36α levels were consistently higher in VNR patients than in VR patients at both time points. Moreover, in both subgroups, IL-36α levels tended to decrease after 48 weeks of BLV treatment, although this reduction was not statistically significant.

In addition, baseline serum IL-36β levels were higher in both VR and VNR patients compared with HBV-monoinfected patients (87.0 [IQR: 73.1–94.4] pg/mL and 85.5 [IQR: 57.3–108.0] pg/mL, respectively; both *p* < 0.0001). At TW48 (Figure 4c), IL-36β levels remained significantly increased in both VR (68.0 [IQR: 58.9–90.9] pg/mL) and VNR patients (59.4 [IQR: 33.6–85.7] pg/mL) relative to HBV-monoinfected patients (both *p* < 0.0001). No significant differences were observed between VR and VNR patients at either BL or TW48. A decreasing trend in IL-36β levels was observed in both VR and VNR patients over the 48-week treatment period, although this trend was not statistically significant.

Finally, IL-36γ levels remained detectable in only a limited number of patients when stratified according to virological response (Figure 4d). At BL, detectable IL-36γ levels were observed in two VR patients (605.6 and 698.2 pg/mL) and in two VNR patients (115.7 and 370.2 pg/mL). At TW48, IL-36γ was detectable in four VR patients (265.8 [IQR: 64.9–435.8] pg/mL) and in only one VNR patient (605.5 pg/mL), while the remaining samples were below the lower limit of detection. Owing to the limited number of detectable values, no formal statistical comparisons between VR and VNR patients were performed.

Analyses of combined response confirmed the same overall pattern (Figure S2a), with significantly higher IL-37 levels in HBV/HDV-coinfected patients, regardless of combined response status, relative to HBV-monoinfected patients at both time points (i.e., BL and TW48). Additionally, no significant differences in IL-37 levels were observed between combined responders (CR) and not combined responders (CNR) at either BL or TW48. Over the 48-week treatment period, IL-37 levels decreased from 239.2 [IQR: 146.8–280.1] pg/mL at BL to 217.2 [IQR: 146.2–299.7] pg/mL at TW48 in CR patients, and from 234.6 [IQR: 188.4–361.3] pg/mL at BL to 190.2 [IQR: 149.7–335.0] pg/mL at TW48 in CNR patients; however, these reductions did not reach statistical significance.

A comparable pattern was observed for IL-36α (Figure S2b). In fact, IL-36α levels were higher in HBV/HDV-coinfected patients irrespective of combined response status compared with HBV-monoinfected patients at both BL and TW48. Notably, a significant difference in IL-36α levels was observed between CR (142.9 [IQR: 100.6–172.0] pg/mL) and CNR (244.5 [IQR: 149.3–298.1] pg/mL) at TW48, with higher levels detected in CNR patients (*p* = 0.04). In addition, opposite temporal trends were observed over the 48-week treatment period, with IL-36α levels tending to decrease in CR patients and to increase in CNR patients; however, these changes were not statistically significant (Figure S2b).

Regardless of combined response status, IL-36β levels were higher in HBV/HDV-coinfected patients compared with HBV-monoinfected patients at both BL and TW48 (Figure S2c). No significant differences in IL-36β levels were detected between CR and CNR patients at either time point, with largely comparable median values at BL (CR: 87.5 [IQR: 83.7–95.2] pg/mL; CNR: 85.0 [IQR: 47.0–96.2] pg/mL) and TW48 (CR: 69.6 [IQR: 56.8–91.6] pg/mL; CNR: 64.8 [IQR: 36.4–77.9] pg/mL). In addition, IL-36β levels showed a similar, non-significant decreasing trend over the 48-week treatment period in both groups.

Finally, analysis based on combined response revealed a distribution of IL-36γ levels comparable to that observed in previous stratifications (Figure S2d). Detectable IL-36γ levels were observed at BL in two CR and two CNR patients, and at TW48 in three CR and two CNR patients, while all remaining samples were below the lower limit of detection.

### 3.5. Association Among Cytokine Levels and HDV RNA

**Figure 5:**
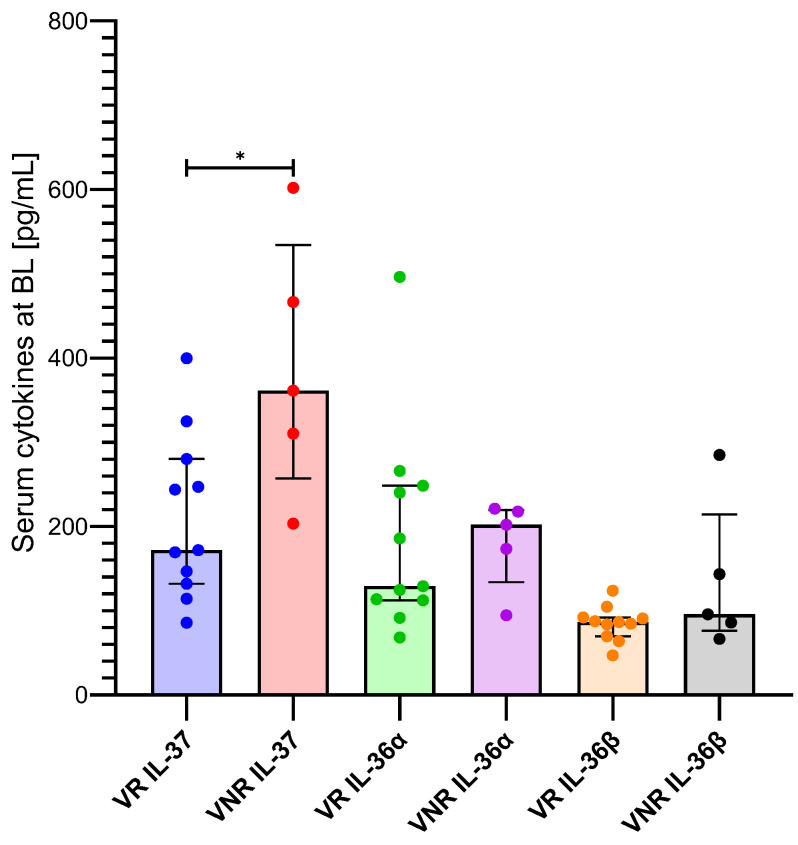
Baseline serum cytokine levels according to virological response at week 96. Serum concentrations of IL-37, IL-36α, and IL-36β measured at baseline (BL) in HBV/HDV-coinfected patients according to virological response at week 96 of bulevirtide treatment. VR, virological responders; VNR, virological non-responders. *p*-values < 0.0332 (*).

Sixteen HBV/HDV-coinfected patients reached week 96 of BLV treatment; among them, 11 achieved a virological response and five did not. When stratified by virological response at TW96, median serum IL-37 levels at BL were significantly lower in patients who achieved virological response than in non-responders (171.9 pg/mL [IQR: 132.1–280.1] vs. 361.3 pg/mL [IQR: 256.8–534.2], *p* = 0.0275). In addition, baseline IL-36α levels were lower in VR than in VNR (129.2 pg/mL [IQR: 112.4–248.4] vs. 202.1 pg/mL [IQR: 134.0–219.5]). In contrast, IL-36β concentrations were comparable between the two groups (86.5 pg/mL [IQR: 69.6–92.1] in VR and 95.7 pg/mL [IQR: 76.3–214.2] in VNR) (Figure 5). At TW48, no significant differences were observed between VR and VNR patients for any of the cytokines analyzed. Specifically, the difference in IL-37 levels between VR and VNR was less pronounced.

**Figure 6:**
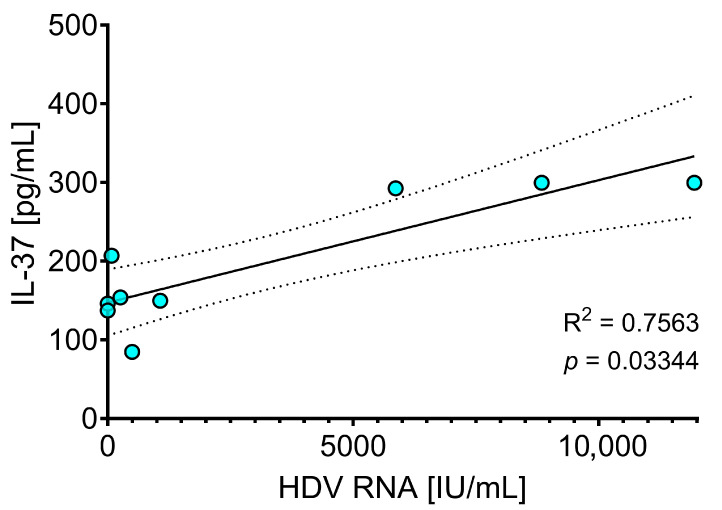
Correlation between serum IL-37 levels and HDV RNA measured by the AltoStar^®^ assay at week 48 of bulevirtide treatment. Correlation of serum IL-37 levels with HDV RNA measured by the AltoStar^®^ assay in HBV/HDV-coinfected patients at week 48 of bulevirtide treatment. Each point represents an individual sample. Regression line, 95% confidence interval, *p*-value, and R^2^ value are shown.

To further explore the relationship between cytokine levels and viral replication, correlations were assessed between serum cytokines and HDV RNA levels measured by both Bosphore and AltoStar^®^ assays at BL and at TW48. Among the cytokines analyzed, only IL-37 showed a significant association with HDV RNA levels at TW48 (Figure 6) when measured by the AltoStar^®^ system, displaying a significant positive Spearman correlation (*p* = 0.033) and a strong linear regression (R^2^ = 0.7563). No significant correlations were observed for IL-36α or IL-36β, nor with HDV RNA levels measured by the Bosphore assay.

## 4. Discussion

Chronic hepatitis D infection represents the most aggressive form of viral hepatitis, characterized by accelerated progression to liver cirrhosis and hepatocellular carcinoma, as well as elevated mortality rates compared to HBV monoinfection [37]. The persistence of viral replication represents a key driver of liver-related morbidity, significantly accelerating progression toward cirrhosis, hepatic decompensation, and mortality [38,39]. However, the clinical use of pegylated interferon has been limited by frequent adverse effects that prevent long-term administration, together with suboptimal virological efficacy [40]. Bulevirtide currently represents a major advance in the therapeutic management of chronic delta hepatitis, owing to its favorable safety profile and suitability for prolonged administration. Nevertheless, real-world data indicate that approximately 50% of patients receiving BLV monotherapy experience virological relapse after treatment discontinuation, with most relapses occurring within the first 24 weeks following drug withdrawal [41]. As a result, real-world evidence on BLV treatment is still evolving, and reliable predictive biomarkers that reflect treatment response and residual viral activity remain lacking. Only lower baseline HDV RNA levels and a sustained duration of HDV RNA undetectability for at least 96 weeks were identified as independent predictors of post-treatment relapse risk [17].

In this study, we investigated the serum profiles of IL-37 and IL-36 isoforms in HBV/HDV-coinfected patients undergoing BLV therapy, comparing baseline and week 48 to evaluate their associations with biochemical, virological, and combined responses. The main finding is that HBV/HDV-coinfected patients display a distinct cytokine signature characterized by persistently elevated IL-37, IL-36α, and IL-36β compared with HBV-monoinfected patients and healthy donors, both at baseline and after 48 weeks of treatment, regardless of response type. Despite partial modulation over time, these cytokines did not fully normalize during therapy, highlighting a sustained inflammatory and immunomodulatory state in chronic delta hepatitis that is not completely reversed by viral entry inhibition with BLV treatment.

In contrast, IL-36γ levels were comparable to those observed in HBV-monoinfected patients and healthy donors and remained largely unchanged during therapy.

Notably, when HDV RNA was quantified using the more sensitive AltoStar^®^ assay rather than the Bosphore assay, IL-37 levels at week 48 showed a significant positive association with residual HDV RNA levels. This finding suggests that IL-37 may reflect ongoing low-level viral replication or intracellular viral persistence rather than categorical treatment response. In patients treated with pegylated interferon-α and ribavirin for HCV, a significant reduction of anti-inflammatory cytokines, including IL-37, has been reported in subjects achieving sustained virological response. In particular, Ding et al. demonstrated that IL-37 levels were significantly elevated in chronic HCV patients compared with healthy controls and markedly decreased after antiviral therapy, independently of treatment outcome, supporting a close link between viral suppression and attenuation of immune activation [42]. This pattern was not observed in BLV-treated patients classified as virological responders. This discrepancy may be explained by the definition of virological response during BLV therapy, which is based on a ≥2 log decline in HDV RNA at 48 weeks and/or HDV RNA levels below the assay limit of detection. As previously demonstrated [43,44], the sensitivity of assays used for HDV RNA quantification varies substantially, and residual HDV RNA may still be present in patients classified as virological responders, potentially sustaining inflammatory cytokine production. Moreover, even in patients achieving virological response, HDV persists within infected hepatocytes and can be transmitted to daughter cells during mitosis, allowing ongoing viral propagation despite BLV treatment. The limited production of IL-36γ observed in BLV-treated patients, with serum levels comparable to those detected in healthy donors, may reflect virus-specific differences in cytokine induction. Indeed, different hepatotropic viruses have been shown to preferentially induce distinct IL-36 isoforms; for example, during Rift Valley Fever virus infection, IL-36γ appears to be the predominantly induced isoform [45].

Overall, serum levels of IL-36 isoforms may serve as relevant surrogate biomarkers of liver immune status during BLV treatment. Previous studies have shown that IL-36 concentrations are higher in patients with hepatocellular carcinoma compared with individuals with chronic hepatitis B, and lowest in healthy controls, with reported serum levels of approximately 200 pg/mL in HCC patients and 70–150 pg/mL in chronic HBV infection. These observations suggest that IL-36 serum concentrations could contribute to the identification of patients at risk for progression toward hepatocellular carcinoma, potentially complementing AFP, which is known to have limited sensitivity and specificity [46,47,48]. In this study, all patients treated with BLV had IL-36β values below 100 pg/mL after 48 weeks of treatment, whereas IL-36α levels below 200 pg/mL were observed only in patients with biochemical response. No cases of hepatocellular carcinoma were observed during follow-up. Further studies are needed to clarify the predictive value of IL-36 isoforms for liver disease progression and HCC development. The three IL-36 isoforms are known to be differentially produced and to exert distinct regulatory effects within the same tissue [49].

Finally, IL-37 levels remained largely unchanged during BLV treatment, consistent with its role as a negative regulator of inflammation through modulation of IFN-γ and TNF-α in the liver, two cytokines critically involved in hepatocyte apoptosis and fibrosis [50,51]. Since HDV RNA often remains detectable at 48 weeks of therapy, ongoing intracellular viral activity may sustain signaling pathways, such as NF-κB and STAT3, which play central role in the regulation of inflammation, apoptosis, and tumor cell proliferation [52,53,54]. In this context, persistent IL-37 expression may represent a compensatory mechanism aimed at limiting excessive inflammatory responses during BLV treatment.

The present study has some limitations. First, it is a retrospective study conducted at a single institution, and the sample size is relatively small. Second, some potential cofactors contributing to systemic and liver inflammation, such as concomitant drug administration, could not be considered in all analyses due to missing data. Future studies should aim to collect a broader set of clinical and therapeutic variables that may influence the efficacy of BLV treatment and to further explore their association with IL-36 and IL-37 production. Additional evidence from larger and prospective cohorts will be required to confirm and extend the present findings.

Despite these limitations, this study provides, to our knowledge, the first evidence of circulating IL-36 and IL-37 cytokines in the serum of patients with chronic delta hepatitis undergoing BLV therapy and offers novel insights into their potential use as complementary biomarkers for monitoring antiviral treatment efficacy, liver disease progression, and the development of hepatocellular carcinoma.

## Figures and Tables

**Table 1:** Baseline characteristics of HBV/HDV-coinfected patients under BLV therapy.

Parameter	N = 22
Age, years	50 (35–68)
Male sex	11 (50.0)
Body mass index	24.5 (23.0–29.7)
Cirrhosis	13 (59.1)
Previous interferon therapy	16 (72.7)
Concomitant NUC therapy	22 (100.0)
ALT, U/L	87.5 (55.3–103.8)
AST, U/L	72 (52.3–88.5)
Albumin, g/dL	4.2 (4.0–4.4)
Bile acids, µmol/L	11.7 (6.4–18.9)
Total bilirubin, mg/dL	0.7 (0.7–0.9)
Platelet count, ×10^3^/µL	113.5 (90.1–190.0)
AFP, ng/mL	4.9 (3.5–6.7)
HDV RNA, Log cp/mL	4.9 (3.9–5.9)
HBV DNA undetectable ^1^	21 (95.5)
HBsAg, Log IU/mL	3.6 (3.3–4.1)
HBcrAg, Log U/mL	3.4 (2.6–4.1)
Anti-HBc IgG, COI	58.5 (40.8–264.2)
HBeAg, positive	1 (4.5)

^1^ HBV DNA < 10 IU/mL. Values are expressed as number (%) or median (IQR1–IQR3), except age (median, min-max). IQR, interquartile range; NUC, nucleos(t)ide-analogue therapy; ALT, alanine aminotransferase; AST, aspartate aminotransferase; AFP, Alpha-Fetoprotein; HDV, hepatitis delta virus; HBV, hepatitis B virus; HBsAg, hepatitis B surface antigen; HBcrAg, hepatitis B core-related antigen; anti-HBc IgG, immunoglobulin G antibody to hepatitis B core antigen; COI, cut-off index. This information was obtained from clinical records.

## Data Availability

The data presented in this study are available on request from the corresponding author.

## Supplementary Materials

The following supporting information can be downloaded at: https://www.mdpi.com/article/10.3390/pathogens15020198/s1, Figure S1: Serum levels of IL-37 and IL-36 family cytokines stratified by cirrhosis status; Figure S2: Serum levels of IL-37 and IL-36 family cytokines stratified by combined response.
