# Supplementary material for: IL-37 and IL-36 Cytokine Profiles in Chronic Hepatitis Delta During Bulevirtide Therapy
Source: Pathogens. 2026 Feb 10;15(2):198. doi: 10.3390/pathogens15020198 (PMC12943429; doi:10.3390/pathogens15020198)
Supplement: Supplementary file 1 [file pathogens-15-00198-s001.zip › Zulian et al_IL37_36_Supplementary.pdf]

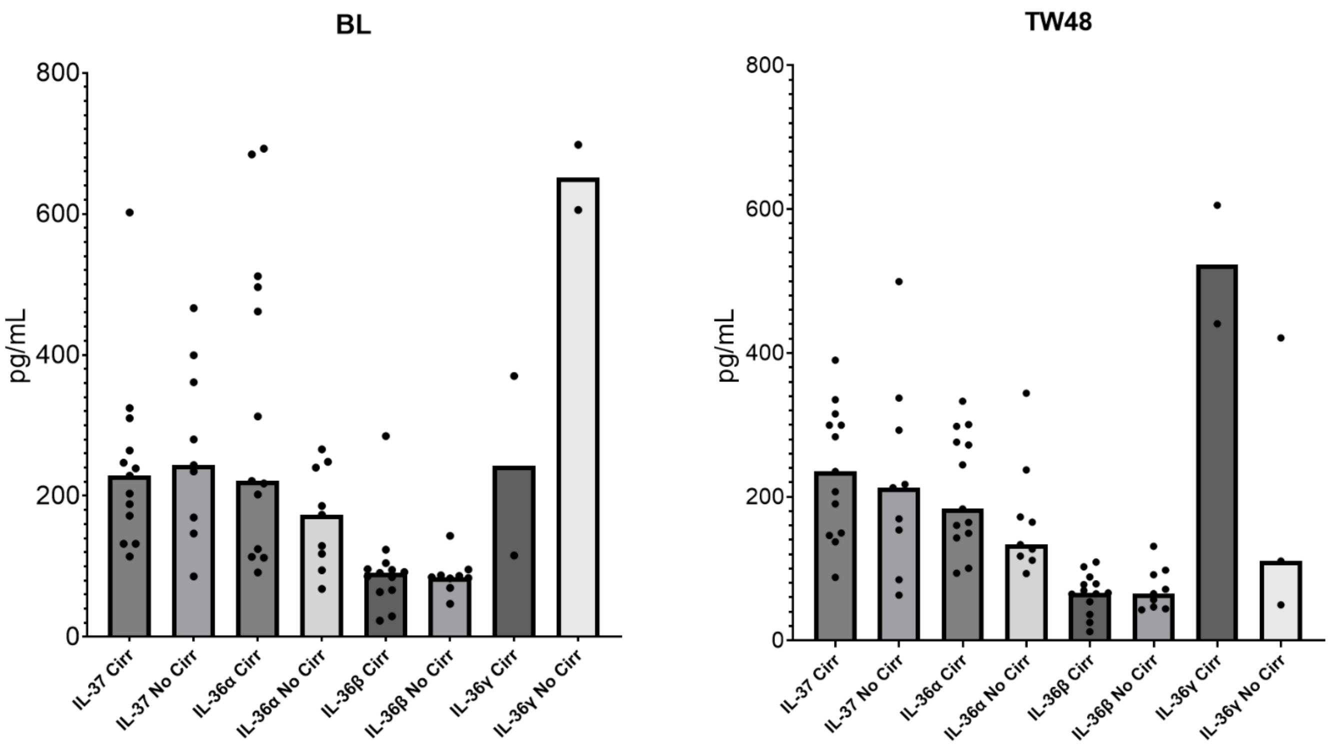

(a) (b)

Figure S1. Serum levels of IL-37 and IL-36 family cytokines stratified by cirrhosis status. Serum concentrations of IL-37, IL-36α, IL-36β, and IL-36γ were measured in HBV/HDV-coinfected patients stratified according to cirrhosis status into cirrhotic (Cirr) and non-cirrotic (No Cirr) at (**a**) baseline (BL) and (**b**) after 48 weeks of bulevirtide treatment (TW48). Individual data points are shown.

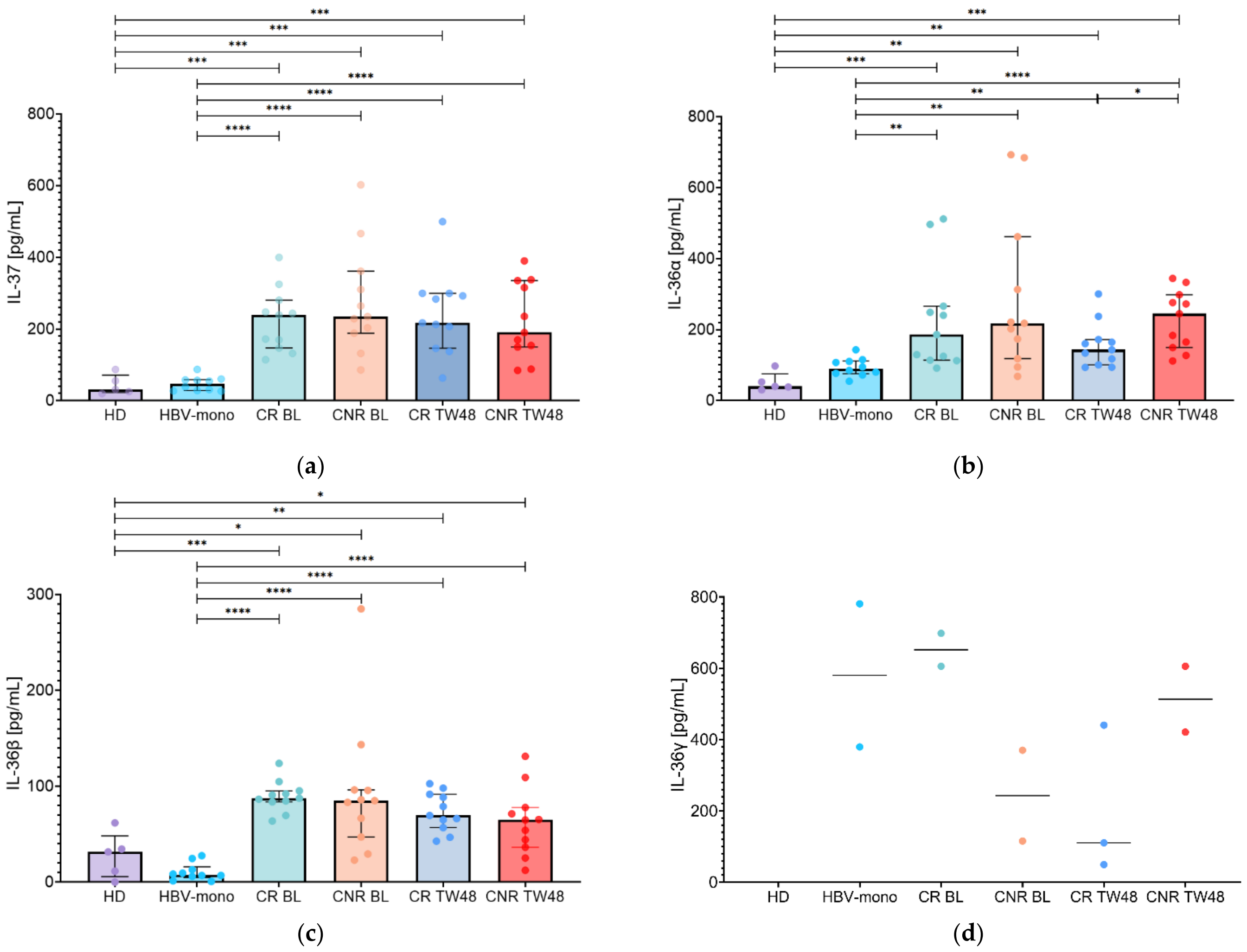

Figure S2. Serum levels of IL-37 and IL-36 family cytokines stratified by combined response. Serum concentrations of (**a**) IL-37, (**b**) IL-36α, (**c**) IL-36β, and (**d**) IL-36γ were measured in healthy donors (HD), HBV-monoinfected patients (HBV-mono), and HBV/HDV-coinfected patients stratified according to combined response into virological responders (CR) and not combined responders (CNR) at baseline (BL) and after 48 weeks of bulevirtide treatment (TW48). Individual data points are shown, with bars representing the median and interquartile range (IQR). P-values are indicated as follows: <0.0332 (*), <0.0021 (**), <0.0002 (***), < 0.0001 (****).
